# The effects of injectable platelet-rich fibrin application on wound healing following gingivectomy and gingivoplasty operations: single-blind, randomized controlled, prospective clinical study

**DOI:** 10.1007/s00784-023-05477-2

**Published:** 2024-01-10

**Authors:** Şeyma Çardakcı Bahar, Nebi Cansın Karakan, Ayhan Vurmaz

**Affiliations:** 1 Department of Periodontology, Gulhane Faculty of Dentistry, University of Health Sciences, Neighborhood of Emrah, Keçiören, Ankara, Turkey; 2 Department of Periodontology, Faculty of Dentistry, Afyonkarahisar Health Sciences University, Afyonkarahisar, Turkey; 3 Department of Biochemistry, Faculty of Medicine, Afyonkarahisar Health Sciences University, Afyonkarahisar, Turkey

**Keywords:** FGF-10, Gingivectomy, I-PRF, VEGF

## Abstract

**Objectives:**

The aim of this study was to evaluate the effects of wound healing using injectable platelet-rich fibrin (IPRF) after gingivectomy and gingivoplasty.

**Materials and methods:**

In this clinical study, 46 systemically healthy patients with chronic inflammatory gingival enlargement were randomly treated with gingivectomy-gingivoplasty + I-PRF (*n*=23) or gingivectomy-gingivoplasty alone (*n*=23). The primary outcome was to evaluate the effect of I-PRF on wound healing over a 3-week follow-up period. Samples collected from gingival crevicular fluid (GCF) were processed using enzyme-linked immunosorbent assay (ELİSA) to measure VEGF and FGF-10 biomarkers. The surgical areas were stained with Mira-2 tone and evaluated in ImageJ. Wound healing was evaluated with Modified Manchester Scar (MMS) scale and Landry, Turnbull, and Howley (LTH) index.

**Results:**

VEGF values of the control group at baseline, week 2, and week 3 were significantly higher than the test group. In weeks 2 and 3, FGF-10 values were found to be significantly higher in the control group than the test group. The amount of staining was found to be significantly higher in the control group than in the test group on days 3, 7, and 14. LTH values of the control group were significantly lower than the test group and MMS values were significantly higher than those of the test group.

**Conclusions:**

I-PRF applications revealed positive effects on epithelial wound healing after gingivectomy and gingivoplasty operations.

**Clinical relevance:**

Platelet concentrates such as I-PRF accelerate wound healing and contribute to the patient’s comfort and quality of life. I-PRF application may have positive effects on wound healing after gingivectomy and gingivoplasty operations.

## Introduction

Gingival enlargement may occur due to many factors, including orthodontic appliances, inflammation, usage of specific drugs, and neoplastic conditions [1]. A gingivectomy operation contributes to periodontal tissue health by removing excess gingival tissues. It improves aesthetics by providing normal physiological contours [2]. Wound healing after a gingivectomy and gingivoplasty is in the form of secondary wound healing [3]. Between 24 and 36 h, epithelial activity at the gingival margin reaches its maximum level. Epithelization of the wound surface is completed within 7–14 days. Complete healing occurs in 30 days with keratinization. The maturation of the connective tissue occurs in 7 weeks [1, 4]. Various products, such as low-dose laser applications, herbal products, hemostatic agents, ozone, antiseptic/antibacterial agents with bioactive material, and platelet concentrates, have been tested to accelerate wound healing after a gingivectomy and gingivoplasty [2, 5].

Platelet-rich fibrin (PRF) is an autologous second-generation blood product rich in leukocytes and platelets. As PRF does not require direct activation by additional factors, such as bovine thrombin or external anticoagulants, both preparation time and cost are significantly lower than platelet-rich plasma (PRP) [6]. For about three decades, PRF has been used for regenerative objectives in dentistry [7]. In 2017, injectable platelet-rich fibrin (I-PRF) was developed by reducing the centrifugation time and speed of PRF and using no-additive plastic centrifuge tubes [8]. Taking advantage of the slower and shorter centrifugation speeds in I-PRF, it can be observed that more regenerative cells contain higher concentrations of growth factors than other PRF formulations using higher centrifugation speeds, as highlighted in previous research [9, 10]. Studies have shown that I-PRF can act as a reservoir of growth factors and lead to migrating key molecules to the application area to improve and support regeneration [11]. Up to the present, various in vitro and in vivo studies have been carried out concerning the role of I-PRF in the enhancement of wound healing, gingival augmentation, acceleration of orthodontic tooth movement, and regeneration of bone, periodontal, and pulp tissues [11–14]. In addition, recent studies have suggested that i-PRF provides a three-dimensional fibrin clot network that includes platelets, leukocytes, type I collagen, osteocalcin, and growth factors, and it acts as a dynamic gel with additional growth factor release for up to 10 days [15, 16].

The researchers hypothesize that I-PRF will accelerate secondary wound healing. According to the researchers’ knowledge, while there has been a previous study investigating the effectiveness of PRF in gingivectomy and gingivoplasty surgeries, there is no study measuring both the wound healing and biochemical effectiveness of I-PRF after gingivectomy and gingivoplasty surgeries [17]. Therefore, this randomized prospective clinical study aimed to evaluate the clinical and biochemical effects of applying I-PRF after gingivectomy and gingivoplasty compared to applying gingivectomy and gingivoplasty alone in systemically healthy individuals with chronic inflammatory gingival overgrowth in the mandibular or maxillary anterior regions. The primary outcome was to evaluate the effect of I-PRF on wound healing over a 3-week follow-up period.

## Materials and methods

In this single-blind, randomized controlled, prospective clinical study, individuals with gingival complaints between August 2021 and March 2022 were diagnosed with chronic inflammatory gingival enlargement as a result of clinical and radiographic examinations. This study was conducted in accordance with the revised Declaration of Helsinki and was approved for human subjects by the Ethical Committee of Afyonkarahisar Health Sciences University (permit number 2021/338). The Afyonkarahisar Health Sciences University Scientific Research Projects Unit supported the study as project number 21.DUS.005. All individuals who participated in the study were informed about the objective and methods of the study and signed informed consent forms. The study was registered at in the US National Institutes of Health Clinical Trials Registry (NCT05871190).

### Determination of sample size and study design

Previous studies on this subject were reviewed, when *α* =0.05 (95% confidence interval), effect size (effect size) was *d*=0.8, and power (1 − *β*) =0.85 (80%) was taken in power analysis, in evaluating the effects of I-PRF applied following gingivectomy and gingivoplasty on wound healing. It was calculated that a minimum of 21 patients should be included in the study for the change in the deepithelized surface area to be 0.60 units in the I-PRF group compared to the control group. Accordingly, considering that there may be missing data, it was decided to select 23 individuals for each group. The study was completed with 46 patients (23 females, 23 males) ages 13–28 years (mean: 16.98 years). The inclusion criteria were as follows: (a) systemically healthy patients ages 13–30 years; (b) no pregnancy or lactation; (c) patients with chronic inflammatory gingival overgrowth in the mandibular and maxillary anterior area ; (d) no clinical attachment and bone loss; (e) not using immunosuppressive agents, systemic corticosteroids, chemotherapy, and/or radiotherapy drugs taken or prescribed 2 months before the study attempt, which may affect the study results, wound healing, and coagulation mechanism; and (f) patients with adequate oral hygiene. The exclusion criteria were as follows: (a) patients who had a history of scaling and root planning in the last 6 months, (b) smokers and alcohol users, (c) those who used drugs that may cause gingival enlargement in the last 6 months, (d) patients with poor communication, and (e) follow-up patients who missed scheduled appointments to collect data.

All patients received initial periodontal therapy (IPT) and oral hygiene instructions. The patients were called again 2 weeks later for control. Gingivectomy and gingivoplasty surgery was planned in patients whose gingival overgrowths were soft and unresistant even after the initial treatment, did not spread to more than six tooth areas, had sufficient attached gingiva, had no attachment loss, and had no intrabony defects [1]. Gingival overgrowth was graded according to the following indices: The buccolingual direction of gingival enlargement was classified according to the index defined by Seymour and later modified by Miranda et al. (MB index) [18]. The vertical direction of gingival overgrowth was also measured according to the index described by Angelopoulos and Goaz and later modified by Miller et al. (GOI index) [19]. Patients with grades other than 0 in both indices were included in the study. A total of two groups, one test and one control, were planned in the study.

Test group: I-PRF is placed on the wound site after a gingivectomy with the conventional method (the procedure of gingivectomy with a #15 scalpel) and closed with a periodontal dressing.

Control group: closure of the wound site with only a periodontal dressing after a gingivectomy with the conventional method.

Investigators’ primary aim in this study was to confirm investigators’ hypothesis that I-PRF accelerates wound healing. Our secondary aim was to see the effect of I-PRF on biochemical parameters.

### Randomization

Sequentially numbered, opaque, sealed envelopes were used for the allocation [20]. An allocation array was created using a computer-generated random list, and sealed, opaque envelopes including the procedures were randomly divided into two groups for each patient by an independent examiner (N.C.K.) with arrays generated using a computer-assisted randomization table (www.randomizer.org; copyright 1997–2011 by Geoffrey C. Urbaniak and Scott Plous). Until the first treatment visit, the physician (Ş.Ç.B.) who was applying and recording the clinical periodontal measurements throughout the study was blinded. All participants were blinded during the practice and control sessions.

### Gingivectomy and gingivoplasty operations

Prior to the procedure, patients were rinsed with mouthwash containing 0.12% chlorhexidine. Surgical operations were carried out with local infiltration anesthesia (Ultracaine D-S; Sanofi Aventis, Germany). A 45-degree inclined external bevel incision was made using a surgical scalpel (Carbon, No. 15) and a gingivectomy blade (Hu Friedy 15/16, Chicago, USA), starting from the distal part of the incision line. The interdental area was addressed using an Orban knife (Hu Friedy 1/2, Chicago, USA), and any remaining granulation tissues were meticulously removed from the surrounding area with the aid of curettes and scissors (Hu Friedy). Subsequently, gingivoplasty was performed using a Kirkland knife (Hu Friedy). This approach was referred to as scalpel (conventional) gingivectomy and gingivoplasty [1, 21].

Considering the results of many studies, it was decided to prefer the conventional method in our study because the cost is lower, the recovery is faster, and the use is practical. After the surgical procedure was completed, the control areas were left to heal spontaneously. I-PRF was applied to the test areas. Surgical areas in the control and test sites were covered with a periodontal dressing (Coepak, Isip, IL, USA).

### Postoperative care and suggestions

Patients were advised to avoid very cold or hot food and beverages to protect the wound area from trauma and to consume very soft foods. They were warned that the periodontal dressing should be in the mouth until the next control day (third day) and that if the periodontal dressing was damaged during that period, they should come to our clinic without delay. The patients were prescribed mouthwash containing 0.12% chlorhexidine and analgesic containing paracetamol (Parol 500 mg). Patients were advised not to brush their teeth in the wound area until the end of the periodontal dressing process (3 days). They were told to provide oral hygiene in the areas where the operation was not performed. Brushing the operation area with a soft toothbrush after the periodontal dressing was removed was recommended.

### Clinical measurements

All operations were performed by a single clinician (Ş.Ç.B) to maintain standardization and apply the single-blind protocol throughout the study. All clinical parameters were measured from the six regions (distobuccal, buccal, mesiobuccal, distopalatinal/lingual, midpalatinal/lingual, mesiopalatinal/lingual) of each tooth using a 0.5-mm-diameter Williams periodontal probe. Clinical measurements included the Löe and Silness gingival index (GI), plaque index (PI) [22], bleeding of probing (BOP) [23], and probing depth (PD). Measurements were made at the baseline (*t*_0_) and on 14 (*t*_1_) and 21 (*t*_2_) days after the gingivectomy and gingivoplasty operations.

### Preparation of I-PRF

**Fig. 1:**
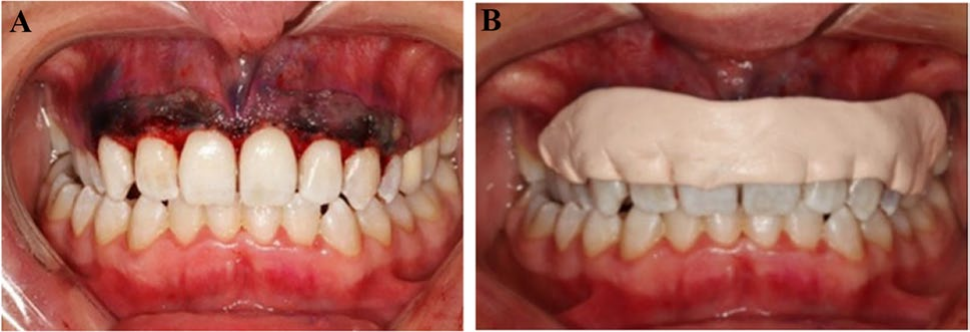
**a** Application of I-PRF to the donor area after gingivectomy and gingivoplasty. **b** Closure of the donor area with a periodontal dressing

The blood samples taken from the patients were collected in 10-ml plastic tubes without anticoagulants. The tubes were centrifuged for 3 min at 2300 rpm (RCF-max (relative centrifugal force) = 509.53*g*) in a centrifuge device (Intraspin centrifugation device) with a 33° rotor angulation with a radius of 53 mm at the clot and 86 mm at the max (PC-O2, Process for PRF, Nice, France) [16, 24]. After centrifugation, the I-PRF in the upper part of the tube was collected via an injector and transferred to a metal godet. It was left for 15–20 min for polymerization of the I-PRF [16]. Afterward, the polymerized I-PRF was applied to the secondary wound, and the operation area was covered with a periodontal dressing (Fig. 1).

### Gingival crevicular fluid sampling

Before gingival crevicular fluid (GCF) samples were taken, saliva contamination was avoided, and the area was isolated with cotton roll pads. GCF samples were collected using sterile paper strips. The strips (Periopaper; Interstate Drug Exchange) were placed in the sulcus until a moderate resistance was felt and left for 30 s [25]. Paper strips contaminated with blood or saliva were discarded. GCF samples were obtained from study participants at the mid-buccal portion of the central teeth (11 or 41), depending on whether the gingivectomy was performed in the upper or lower jaws. This procedure was repeated in the same regions before the operation (baseline) and on days 14 and 21. Samples were stored in Eppendorf tubes at −20°C until further processing was carried out [25]. The samples were analyzed using the ELISA test. The test followed the manufacturer’s instructions, and the mean VEGF and FGF-10 levels were calculated for each operation site.

### Biochemical analysis of GCF samples

GCF samples of the test and control groups were placed in Eppendorf tubes and stored until the study day at −20°C. A phosphate buffer saline (PBS) solution (pH: 7.4) of 0.2 ml was added to the GCF samples. One minute ultrasonication at 20,000 rpm was performed with a Hielscher (Germany) sonicator.

The GCF samples were then centrifuged at 10,000*g* for 15 min, and the supernatant was used. FGF-10 and VEGF levels were studied at 450 nm with ELISA (Bioassay Technology Laboratory, Shanghai/China) kit and Thermo Scientific Multiskan FC Microplate (Thermo Fisher Scientific Instruments Co. Ltd. Shanghai/China) reader at 450 nm. The results were expressed as nanograms per liter.

### Clinical follow-up of patients

Patients who were included in the study and underwent gingivectomy and gingivoplasty were called back for follow-up appointments on days 3, 7, 14, and 21. The parameters evaluated in the control sessions were as follows:

1. Evaluation of wound epithelialization with a Mira-2 tone solution.
2. LTH wound healing index (valuation of soft tissue healing).
3. MMS scale (evaluation of soft tissue healing).
4. Assessment of pain.

### Taking photos of operation areas and evaluation of epithelialization of the wound area

**Fig. 2:**
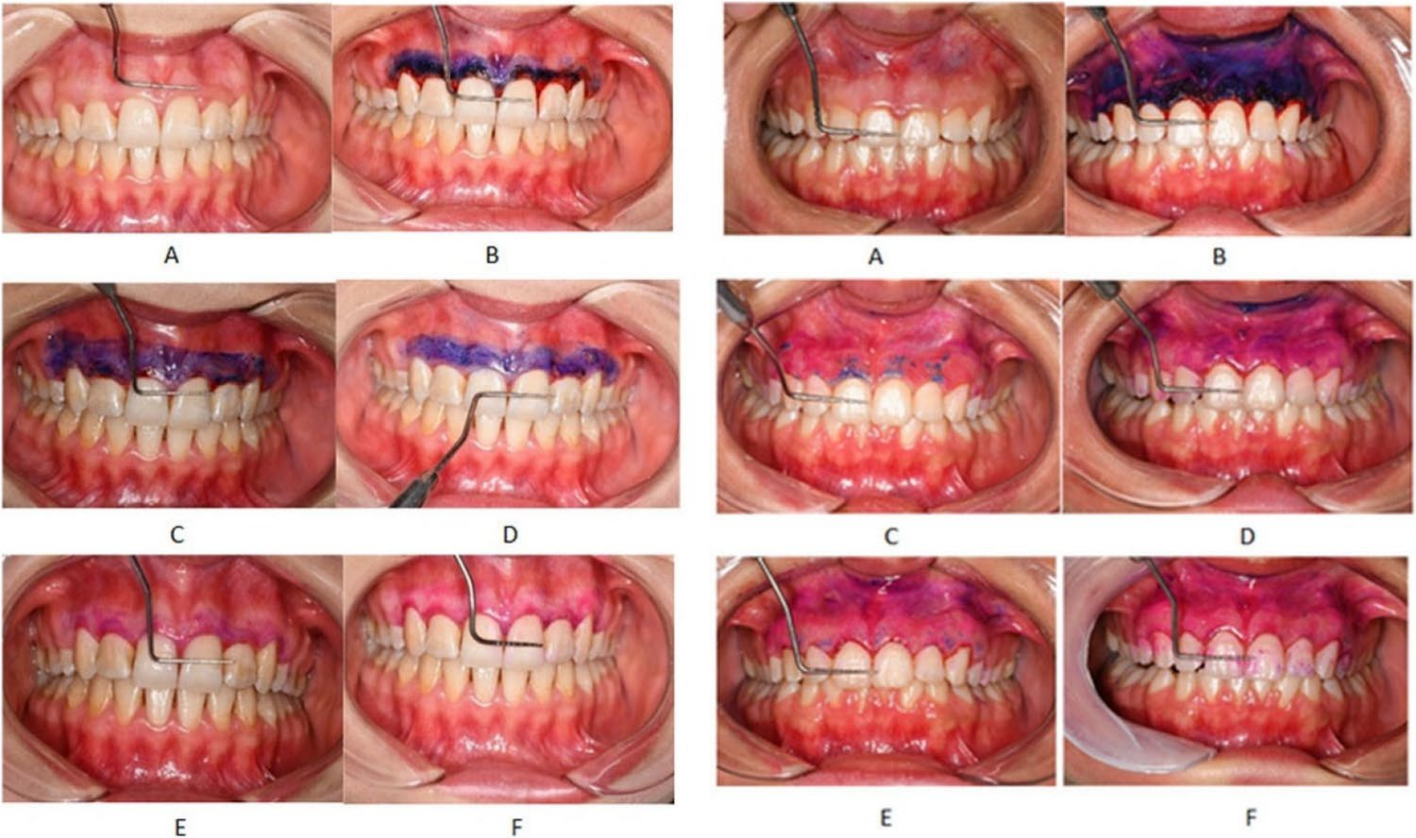
Intraoral photographs of the control group and test group patients and calculation of stained area in ImageJ program. (**A**) Before the operation. (**B**) Immediately after the operation. (**C**) 3rd day after the operation. (**D**) 7th day after the operation. (**E**) 14th day after the operation. (**F**) 21st day after the operation

**Fig. 3:**
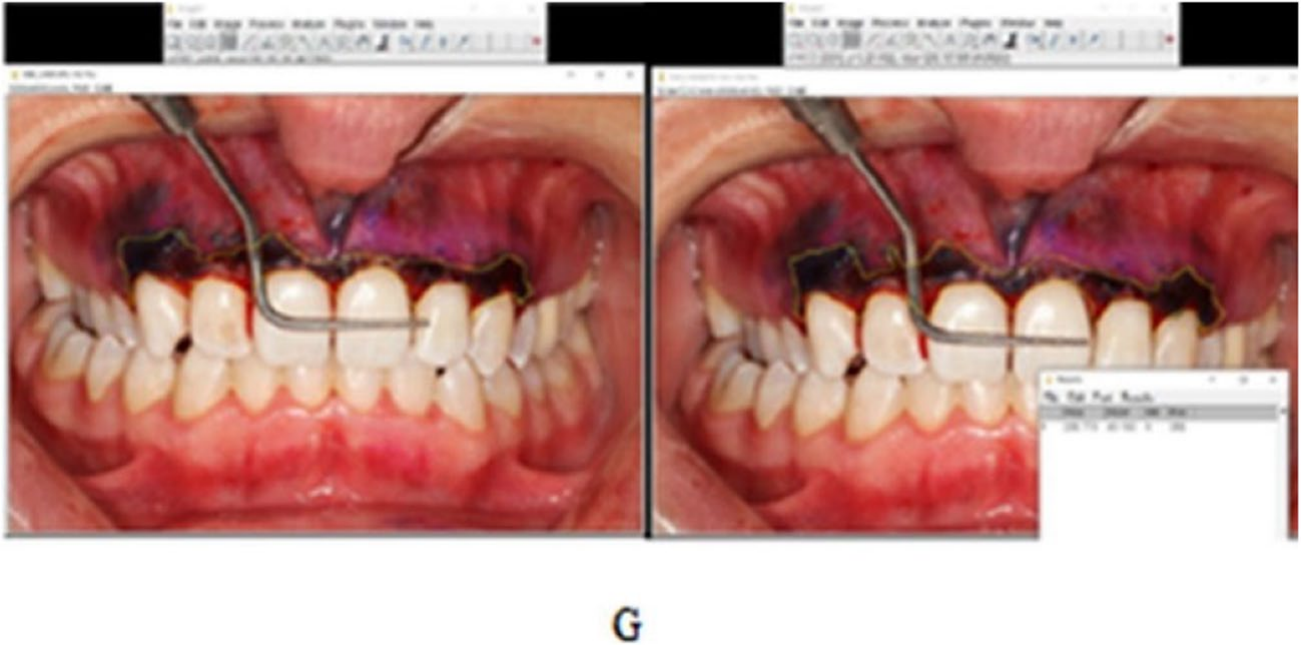
Calculating the stained area in the ImageJ program

Mira-2 tone solution, which is a plaque-disclosing agent, was used to measure wound area epithelialization on postoperative days 0, 3, 7, 14, and 21 (Fig. 2). This solution has been used in where epithelization is lacking [26]. The areas of abrasion and the level of epithelialization during post-operative healing were evaluated. Wound surface epithelialization in the obtaining photographs was evaluated using the ImageJ software program (National Institutes of Health, USA ImageJ 1.48V) (Fig. 3). Photo size was scaled using a 10-mm Williams periodontal probe during photographing. To ensure standardization, all photographs were taken by the same person with the same camera at the same angle, distance (20 cm), and light values (ISO-800) (Nikon D7500). The actual intraoral dimensions (mesial to distal) of the maxillary right central incisor in each patient were measured and compared with the dimensions of the right central incisor in the images, and the ratio between actual and photographic size was used to calibrate the images. In addition, wound surface epithelialization area measurements were repeated at 1-week intervals over photographs of 10 patients who had undergone gingivectomy and gingivoplasty and were not included in the study for the calibration of the person who made the measurements [26].

### LTH index

Wound healing was evaluated by using the LTH index, which classifies the healing period according to redness, granulation tissue, presence of bleeding, suppuration, and epithelialization. In this index, recovery is scored between 1 (very poor recovery) and 5 (excellent recovery) [27]. It was evaluated in both the control and test groups on days 3, 7, 14, and 21.

### MMS scale

The MMS scale was used to evaluate the contour of the wound area, distortion status, and color match. A low total score indicates weak healing, and a high score indicates good healing [28]. It was evaluated in both the control and test groups on postoperative days 3, 7, 14, and 21.

### Biochemical analysis of GCF samples

GCF samples of the test and control groups were placed in Eppendorf tubes and stored until the study day at −20°C. A phosphate buffer saline (PBS) solution (pH: 7.4) of 0.2 ml was added to the GCF samples. One minute ultrasonication at 20,000 rpm was performed with a Hielscher (Germany) sonicator.

The GCF samples were then centrifuged at 10,000*g* for 15 min, and the supernatant was used. FGF-10 and VEGF levels were studied at 450 nm with ELISA (Bioassay Technology Laboratory, Shanghai/China) kit and Thermo Scientific Multiskan FC Microplate (Thermo Fisher Scientific Instruments Co. Ltd. Shanghai/China) reader at 450 nm. The results were expressed as nanograms per liter.

### Assessment of pain

İnvestigators’s study used a Visual Analog Scale to measure pain levels during the post-operative recovery period. Patients were asked to score their current pain status between 0 and 10 on the same days, and on days 3 and 7 after the operation (0 = no pain, 10 = worst pain) [29].

### Statistical analysis

IBM SPSS.25 program was used in all statistical analyses. Normality assumptions were examined with the Shapiro–Wilk test. An independent sample *t*-test was used for inter-group statistical comparisons of the parameters showing normal distribution, and the Mann–Whitney *U* test was used for inter-group statistical comparisons of parameters showing non-normal distribution. In intragroup comparisons, the one-way analysis of variance or Friedman test was used for repeated measurements according to the assumption of normality, and post hoc tests with Bonferroni correction were used in case of significant differences. Correlations between categorical variables were examined by chi-square analyses. For all tests used, values of *P* <0.05 were considered statistically significant

## Results

The clinical part of our study started in August 2021 and ended in March 2023 when we reached the desired sample size. No participant was excluded from the study. Forty-six individuals participated in all analyses. A total of 46 patients, 23 (50%) in the control group and 23 (50%) in the test group, were included in the study. Twenty-three (50%) were women. The mean age of the patients was 16.98 ± 3.27 years. PI, GI, PD, and BOP scores at baseline, day 14, and day 28 are presented in Table 1. The BOP levels (%) of the control group at the beginning and on day 21 were found to be significantly higher than the test group (*p*<0.05) (Table 1). VEGF scores in the control group were found to be significantly higher compared with the test group on the baseline, day 14, and day 21 (*p*<0.05). While the FGF-10 scores in the control group in weeks 2 and 3 were significantly higher than in the test group, the initial FGF-10 score was significantly lower than in the test group. No significant difference was found in the in-group comparisons (Table 2). In the control group surface area staining levels on day 3, week 1, and week 2 were higher than those of the test group. The in-group comparison of group surface area staining levels is presented in Table 3. The MMS levels of the control group were significantly higher on days 3, 7, 14, and 21 compared to the test group. LTH levels of the control group were significantly lower on days 3, 7, 14, and 21 compared to the test group. In terms of MMS and LTH levels, there was a significant difference in both groups on days 3, 7, 14, and 21 in the in-group comparisons (Table 4).

**Table 1:** Comparison of PI, GI, PD, and BOP according to groups and time

Parameters	Baseline		2nd week		3rd week		*p*
	Mean ± SD		Mean ± SD		Mean ± SD		
PI Control	*0.83 ± 0.31*^*¥*^		0.63 ±0.28		*0.63 ± 0.23*^*¥*^		***p*=0.011**^**b**^
Test	0.89 ± 0.29		0.76 ± 0.23		0.75 ± 0.29		*p*=0.100^b^
Total	0.86 ± 0.30		0.69 ± 0.26		0.69 ± 0.27		
*p*		*p*=0.517^a^		*p*=0.082^a^		*p*= 0.138^a^	
GI							
Control	*1.53 ± 0.30*^*¥*^		1.35 ± 0.24		*1.22 ± .021*^*¥*^		*p*=0.001*
Test	*1.39 ± 0.23*^*¥*^		1.33 ± 0.20		*1.16 ± 0.20*^*¥*^		*p*=0.039*
Total	1.46 ± 0.28		1.34 ± 0.22		1.19 ± 0.21		
*p*		*p*=0.103^ǂ^		*p*=0.733^ǂ^		*p*=0.397^ǂ^	
PD							
Control	*2.05 ± 0.51*^*¥*^		*1.22 ± 0.19*^*¥*^		*1.29 ± 0.30*^*¥*^		*p*<0.001*
Test	*1.74 ± 0.45*^*¥*^		*1.22 ± 0.13*^*¥*^		*1.23 ± 0.25*^*¥*^		*p*<0.001*
Total	1.90 ± 0.50		1.22 ± 0.16		1.26 ± 0.27		
*p*		***p*=0.032**^**ǂ**^		*p*=0.363^ǂ^		*p*=0.434^ǂ^	
BOP							
Control	*46.19 ± 19.64*^*¥*^		*33.78 ± 15.19*^*¥*^		*21.23 ± 13.49*^*¥*^		*p*<0.001*
Test	*32.10 ± 20.16*^*¥*^		*28.50 ± 16.90*^*¥*^		*13.00 ± 6.96*^*¥*^		*p*=0.002*
Total	39.14 ± 20.93		31.14 ± 16.11		17.11 ± 11.40		
p*		***p*=0.019**^**ǂ**^		*p*=0.242^ǂ^		***p*=0.019**^**ǂ**^	

Values in bold are statistically significant. There is a significant difference between the values written in italics and carrying the ¥ symbol in the intragroup comparison

*PI* plaque index, *GI* gingival index, *PD* probing of depth, *BOP* bleeding of probing

^a^Independent sample *t* test

^b^One-way ANOVA and post hoc test

^ǂ^Mann–Whitney *U* test; *Friedman and post hoc test

**Table 2:** Comparison of VEGF and FGF10 by groups and time

Parameters	Baseline		2nd week		3rd week		
	Mean ± SD		Mean ± SD		Mean ± SD		*p*
VEGF							
Control	13236.13 ± 2223.54		13590.78 ± 2883.74		14069.57 ± 1908.60		*p*=0.878**
Test	10143.70 ± 2036.89		10081.48 ± 1685.56		9649.22 ± 2303.02		*p*=0.260**
Total	11689.91 ± 2624.77		11836.13 ± 2932.88		11859.39 ± 3060.61		
p*		*p*<0.001*		*p*<0.001*		*p*<0.001*	
FGF-10							
Control	1095.56 ± 192.08		1137.31 ± 195.16		1061.13 ± 195.34		p= 0.568**
Test	1145.00 ± 1774.01		862.28 ± 248.15		882.92 ± 273.34		p= 0.568**
Total	1120.28 ± 1247.90		999.79 ± 260.87		972.02 ± 251.59		
p*		*p*<0.001*		*p*<0.001*		*p*=0.010*	

Values in bold are statistically significant

*VEGF* vascular endothelial growth factor, *FGF-10* fibroblast growth factor 10

*Mann–Whitney test; **Friedman test

**Table 3:** Evaluation of surface area staining levels between and within groups

Parameter surface area Staining levels	Baseline	3rd day	1st week	2nd week	3rd week	
	Mean ± SD	Mean ± SD	Mean± SD	Mean±SD	Mean±SD	p**
Control	112.74±9.21	74.00 ±6.62	44.90 ±6.23	16.84±2.60	0.97 ± .44	***p*<0.001**
Test	126.96±13.21	42.66 ± 2.57	23.34 ±2.83	5.94 ± 1.56	0.80 ± .34	***p*<0.001**
Total	119.85±13.36	58.33±16.60	34.12±11.90	11.39±5.90	0.89 ± .40	
p*	***p*<0.001**	***p*<0.001**	***p*<0.001**	***p*<0.001**	*p*= 0.111	

Values in bold are statistically significant

*Mann–Whitney test; **post hoc and Friedman test

**Table 4:** Comparison of MMS-total and LTH by groups and time

Parameters	3rd day		1st week		2nd week		3rd week		
	Mean ±SD		Mean ±SD		Mean ±SD		Mean ±SD		p**
MMS-total									
Control	4.83 ± 1.07		3.91 ± 1.12		3.09 ± 0.85		2.43 ± 0.95		***p*<0.001**
Test	3.30 ± 0.63		2.26 ± 0.75		1.48 ± 0.51		0.74 ± 0.54		***p*<0.001**
Total	4.07 ± 1.16		3.09 ± 1.26		2.28 ± 1.07		1.59 ± 1.15		
p*		***p*<0.001**		***p*<0.001**		***p*<0.001**		***p*<0.001**	
LTH									
Control	1.87 ± 0.63		2.52 ± 0.67		3.00 ± 0.67		3.57 ± 0.66		***p*<0.001**
Test	2.74 ± 0.45		3.30 ± 0.56		3.87 ± 0.34		4.43 ± 0.51		***p*<0.001**
Total	2.30 ± 0.70		2.91 ± 0.72		3.43 ± 0.69		4.00 ± 0.73		
p*		***p*<0.001**		***p*<0.001**		***p*<0.001**		***p*<0.001**	

Values in bold are statistically significant

*MMS* Modified Manchester Scar scale, *LTH* Landry, Turnbull, Howley Index

*Mann–Whitney test; **Friedman and post hoc test

## Discussion

In this study, we investigated the effects of I-PRF application on wound healing after gingivectomy and gingivoplasty in patients with chronic gingival overgrowth and the results of this study showed that the acceleration in wound healing due to the application of I-PRF after gingivectomy and gingivoplasty was statistically significant. VEGF scores in the control group were found to be significantly higher compared with the test group on the baseline, day 14, and day 21. While the FGF-10 scores in the control group in weeks 2 and 3 were significantly higher than in the test group, the initial FGF-10 score was significantly lower than in the test group. No significant difference was found in the in-group comparisons. On all follow-up days, MMS levels of the control group were significantly higher than the test group, while LTH levels were significantly lower than the test group.

Wound healing is a complex process that involves a coordinated series of events [30, 31]. In wound healing, the aim is to re-establish tissue integrity, oxygenate the tissue, and regain functional and aesthetic patient comfort. Various methods and materials are available to accelerate wound healing, reduce bleeding and pain, and increase patient comfort after gingivectomy and gingivoplasty operations. Recent studies have obtained favorable results on the effects of PRF derivatives on secondary wound healing [16, 17, 32].

I-PRF was preferred in our study because it is completely autogenous and does not contain anticoagulants known to negatively affect wound healing. Studies have reported that I-PRF contains VEGF, EGF, IGF, platelet-derived growth factor (PDGF), and FGF; however, there is no clear conclusion about how many types of growth factors I-PRF contain. Thanks to the type 1 collagen and growth factors it contains, I-PRF is involved in tissue reconstruction, bone remodeling, and regulation of the wound healing process [33, 34]. Wang et al. compared I-PRF and PRP on the movements of fibroblasts obtained from rough and smooth titanium implant surfaces. I-PRF was found to accelerate osteogenic differentiation by providing a higher level of cell migration compared to PRP [13]. A study by Miron and Fujioka-Kobayashi on PRP and its derivatives found that PRP released significantly higher and earlier (15 min) than I-PRF, but PDGF-AA, PDGF-AB, EGF, and IGF-1 all showed higher levels of release in I-PRF compared to PRP. Interestingly, the total growth factor release of PDGF-BB, VEGF, and TGF-β1 is significantly higher in PRP compared to i-PRF. The major advantage of I-PRF is that it remains a 100% autologous product with the advantage of forming a fibrin clot while maintaining growth factor release comparable to PRP [33].

Periodontal parameters of patients were recorded just before the gingivectomy procedure and 14 and 21 days after the I-PRF application, and as a result, it was found that the gingivectomy procedure was effective in reducing GI, PD, and BOP scores in the current study. Pilloni et al. and Lione et al. applied only IPT to some of their patients with gingival enlargement, while others underwent IPT+gingivectomy. Similar to our study, they found reductions in clinical parameters in patients who underwent IPT+gingivectomy compared to those who received only initial periodontal treatment [35, 36]. In addition, the percentage of bleeding on probing on day 21 of the control group was found to be significantly higher than the test group in the present study. This is thought to be related to the fact that I-PRF positively affects wound healing and reduces inflammation.

FGF-10 (KGF-2) is a growth factor that can regulate the proliferation and differentiation of keratinocytes, which has an important role in maintaining normal tissue structure and promoting wound healing [37]. In our study, FGF-10 scores in the control group were found to be significantly higher than the test group in the second and third weeks. It was observed that the initial FGF-10 score was significantly lower than the test group. In the animal study conducted by Beer et al., it was mentioned that FGF-10 is high in the wound area and the level may increase depending on the wound [38]. Robson et al. reported in their study that topical FGF-10 accelerated wound closures in chronic venous ulcers [39]. On the other hand, Cai et al. found in a study they conducted in rats that topical KGF-2 accelerated corneal epithelial wound healing, inhibited corneal neovascularization, and reduced inflammation, stromal edema, and fibrosis [40]. In line with these results, FGF-10 holds great promise in promoting re-epithelialization and wound healing in many tissue injury models.

Angiogenesis is a critical stage of wound healing, and its failure results in chronic wounds. VEGF, an important dynamic molecule of angiogenesis, is involved in different stages of wound healing [41]. VEGF scores in the control group were found to be significantly higher compared with the test group on the baseline, day 14, and day 21 (*p*<0.05). No significant difference was found in the in-group comparisons. Johnson and Wilgus found in their study that high VEGF levels are generally associated with immature, poorly perfused wounds [42]. In a systematic review on the healing of venous leg ulcers [VLU], it was reported that IL-1, IL-6, IL-8, TNF, and VEGF levels increased in the nonhealing phase, but decreased when it was decided that the VLUs were healed. VEGF increased at early time points after a skin injury, reached a maximum on day 3, decreased by day 13, and returned to its normal value by day 21 [43]. Our study results were found to be compatible with the literature. This may be due to the lack of statistical differences in intra-group comparisons.

In studies investigating wound healing following gingivectomy operations, one of the most crucial criteria is the level of reepithelialization in the surgically treated area. To ensure greater objectivity compared to other methods, in our study, Mira-2 tone solution was utilized for the measurement of reepithelialization levels, aligning with the literature [26, 44]. By transferring the standardized photographs taken after the solution was applied to an image analysis program (ImageJ 1.31o, National Institutes of Health, Bethesda, MD), they calculated the dark-colored areas in the computer environment. The results of the calculations made by the program were accepted and calibrated in international evaluations [26]. According to the image analysis results of our study, non-epithelialized areas regularly decreased until day 21 after the gingivectomy operation in both groups. This decrease was statistically significant in both groups; on days 14 and 21, the majority of epithelialization was completed without any problems in the test and control groups. In comparing the groups, the non-epithelialized areas measured in the test group were significantly lower on days 3, 7, and 14 compared to the control group, but there was no significant difference between the groups on day 21. The reason for less staining in the test group is that epithelialization was higher due to the effect of I-PRF. On day 21, the completion of keratinization in both groups can be considered as the reason for the absence of a significant difference between the groups. In the study conducted by Bozkurt et al. [17], they found a significantly lower rate of staining in the groups where PRF and its derivatives were applied compared to the control group. This result is consistent with our study.

After gingivectomy and gingivoplasty surgeries, the bleeding status of the wound area, granulation tissue, color, and presence of suppuration were evaluated using the LTH index [27]. It was observed that LTH values in the test group were higher in all control sessions (days 3, 7, 14, and 21). Similarly, Bozkurt et al. [17] used the LTH index while measuring the effect of different platelet concentrates (PRF, CGF, and autogenous fibrin glue (AFG)) on wound healing in the operation areas after gingivectomy and gingivoplasty. They found significantly higher LTH index values in the test groups compared to the control group in evaluations on days 7, 14, and 28. However, Kızıltoprak et al. used the LTH index while evaluating the effect of AFG and i-PRF on wound healing in the donor area in the free gingival graft operation and found higher LTH index values on AFG-applied surfaces in all control sessions (days 3, 7, 14, and 28, and month 3) compared to the control group and I-PRF-applied surfaces [16].

The MMS scale shows the degree of repair in the wound by evaluating the contour, distortion, and color of the wound [28]. In the present study, the MMS score was found to be higher in the test group than in the control group in all control sessions (days 3, 7, 14, and 21) [16]. Similarly, in the split-mouth study conducted by Samani et al., in which the effect of PRP on wound healing in the donor area was investigated after the free gingival graft operation, it was observed that MMS scores were significantly better in the PRP group at all time intervals and especially on the seventh day [45]. In the study by Kızıltoprak et al., when examining the MMS scores on the third and seventh days, it was observed that the AFG and I-PRF groups were significantly better than the control group [16].

Considering the limitations of the study, the inability to obtain gingival homogenate for pathological evaluation from the operation area can be seen as a deficiency to support the results. More frequent GCF samples could not be taken due to the difficulty in obtaining healthy samples from blood and saliva in the region. For this reason, we received the first samples on day 14 instead of day 3. Moreover, to ensure standardization, all photographs were taken by the same person with the same camera at the same angle, distance (20 cm), and light values (ISO 800) (Nikon D7500), but human errors are still possible. Therefore, the patient’s head would be better positioned and stabilized with a panoramic imaging unit, such as in orthopantomography, along with a digital camera mounted on a tripod.

## Conclusion

In this study, it was shown that I-PRF positively affected wound healing and epithelialization following gingivectomy and gingivoplasty operations according to the Mira-2 tone staining, MMS scale, and LTH index. For the purposes of learning the effects of I-PRF on clinical and biochemical parameters more comprehensively, further studies are needed in which these parameters are evaluated at more frequent intervals.

## Data Availability

The full trial protocol relevant to the study is available from the corresponding author upon request.
